# Exercise Duration Modulates Cortisol Release and Chronic Cortisol Exposure Jeopardises T Cell Effector Functions

**DOI:** 10.1111/imm.70028

**Published:** 2025-08-12

**Authors:** Thy Viet Luu, Line Fleischer Hach, Tina Seremet, Katharina Leuchte, Per thor Straten, Gitte Holmen Olofsson

**Affiliations:** ^1^ National Center for Cancer Immune Therapy (CCIT‐DK), Department of Oncology University Hospital Herlev Herlev Denmark

**Keywords:** cancer, neuroimmunology, regulation/suppression, T cell

## Abstract

Psychological stress has been linked to increased incidence and mortality of cancer. During stress, cortisol is released into circulation and regulates cellular processes including immune activity by acting on glucocorticoid receptors (GCRs) expressed by target cells. Chronic stress‐induced cortisol has been suggested to promote tumour progression and compromise the efficacy of cancer treatments. Conversely, cortisol is also transiently secreted during exercise. Although exercise has been suggested to have beneficial effects against cancer, the impact of exercise‐elevated cortisol on immune cell functions remains poorly understood. Here we studied the dynamics of cortisol secretion following exercise and how cortisol affects effector functions of T cells in the context of acute versus chronic stress. We show that 40 min of acute, high‐intensity exercise in healthy adults significantly increased stable circulating cortisol levels whereas a 5‐min sprint failed to. Acute exposure to cortisol for 4 h showed no negative effects on the proliferation, cytokine release, or killing activity of human CD3^+^ T cells. In contrast, chronic cortisol dampened these T cell effector functions. Furthermore, chronic cortisol exposure induced the proliferation of several cancer cell lines. Our findings highlight the opposing effects of cortisol during acute stress, such as exercise, compared to chronic stress, on cancer cells and T cells. This suggests an important potential in targeting cortisol signalling to enhance cancer immunotherapy.

## Graphical abstract

Cortisol has been linked to poor cancer prognosis but can also be released during exercise. This study shows that transient cortisol exposure, as seen with exercise, does not impair T cell effector functions. In contrast, chronic cortisol signalling suppresses T cell functionality and promotes cancer cell proliferation, highlighting the importance of cortisol duration in regulating T cell antitumor activity.

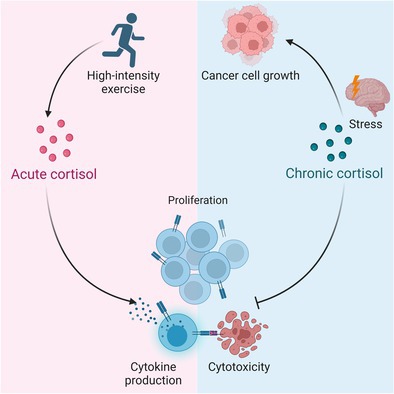

## 1 Introduction

Stress has been suggested to associate with negative clinical outcomes in several cancers [1, 2]. In response to stress, the hypothalamic–pituitary–adrenal (HPA) axis is activated, which leads to the release of glucocorticoids (GCs), mainly cortisol in humans, from the adrenal cortex. GCs act by binding to glucocorticoid receptors (GCRs) expressed in the cytosol of nearly all cells in the human body [3]. Upon binding, this receptor–ligand complex translocates to the nucleus and regulates the expression of target genes through the activation or repression of their transcriptional activity. The direct effect of GCs on tumour cells can vary, but they are mainly suggested to induce genomic instability, metastasis, and angiogenesis of cancer cells [4]. Furthermore, chronic stress‐induced GCs have been shown to promote tumour development and are associated with poor prognosis in cancer patients [5]. Stress‐elevated levels of GCs are also suggested to attenuate antitumor immune responses and compromise the efficacy of anticancer therapies [5]. There is evidence that patients with non‐small‐cell lung cancer under treatment with GCs showed shorter survival and reduced responsiveness to immune checkpoint inhibitors [6].

In many cancer types, regular exercise has been suggested to lower incidence, risk of recurrence and mortality [7]. The immune system is suggested to play a profound role in these effects through leukocytosis, a process where major leucocyte subtypes are mobilised following exercise‐induced stimuli. During exercise, signalling molecules including interleukin‐6 (IL‐6), IL‐7, IL‐15, catecholamine, and cortisol are released into the bloodstream, along with changes in the circulation of immune cells [8]. These cells, mostly T and natural killer (NK) cells, have been shown to infiltrate tumours and reduce tumour growth in mice [9, 10]. Unlike psychological distress where the levels of cortisol remain elevated, the exercise‐induced cortisol is known to be relatively transient and return to baseline levels during the recovery from exercise, representing acute stress [11]. This is due to the negative regulation of the HPA axis by excessive levels of cortisol. However, findings on the increase in cortisol levels vary depending on the intensity and duration of the exercise session. Furthermore, despite being one of the hormones associated with exercise‐induced leukocytosis, the effect of cortisol on immune cell functions during exercise is largely unknown.

Overall, GCs are pivotal stress‐induced hormones that bridge the nervous and the immune systems—in the context of both exercise and cancer. Stress involves the release of several hormones, but the immunoregulatory role of GCs alone is not clearly understood. GCs suppress the transcription of pro‐inflammatory genes in innate immune cells as well as genes involved in T cell activation, antigen presentation, and co‐stimulation [5, 12]. Therefore, GCs are best known for their immunosuppressive properties and are mainly used in the clinic for inflammation treatment. However, a growing number of studies have suggested that endogenous GCs like cortisol can both promote or inhibit immune responses depending on the concentration, duration of action, and target cells [13, 14]. Therefore, one could speculate that the impact of cortisol on immune cells during acute stress, such as exercise, differs from the effect in chronic stress situations, such as cancer. In this study, we investigate the connection between cortisol and physical activity, as well as the impact of cortisol on T cell effector function. We hypothesise that acute levels of cortisol do not hamper effector functions of T cells, whereas chronic cortisol signalling dampens T cell activities.

## 2 Materials and Methods

### 2.1 Experimental Design and Sample Collection of Exercise Trials

Healthy participants aged 25–65 performed one of two acute exercise programmes. For the first exercise protocol, six healthy participants (four males and two females) ran up and down two flights of stairs for 3.5 min followed by 1.5 min of high‐intensity level ground running. No measurements of exercise intensity were performed, but the participants were encouraged to exercise at high intensity—that is, “run as fast as you can”. Blood samples were collected directly before and within 2 min after completing the exercise (Vacuette EDTA, Greiner bio‐one).

The second sample group is derived from the INHALE study (NCT05826496). Nine healthy participants (six males and three females) were recruited. Physical activity history was assessed using the International Physical Activity Questionnaire (IPAQ), with a mean activity level of 2844.8 MET‐minutes per week. Exclusion criteria included beta‐blocker or corticosteroid use. Before starting high‐intensity exercise training (HIIT), participants completed a cardiopulmonary exercise test (CPET) on a bicycle ergometer (Motion cycle 200 med, Emotion Fitness GmbH & Co) to determine maximum power output, aerobic capacity (VO_2_ peak) and respiratory exchange rate (RER). Alcohol, non‐prescription medication, and strenuous exercise were avoided for 24 h before CPET and HIIT. At the start of HIIT, baseline blood samples were taken by venipuncture (Vacuette Serum, Greiner bio‐one). The warm‐up consisted of 5 min on a bicycle ergometer (Motion cycle 200 med, Emotion Fitness GmbH & Co) followed by 5 × 2‐min circuit training on: (1) an air bike ergometer (ReNegaDE AIR BIKE C2), (2) a ski ergometer (CONCEPT 2 Deutschland), (3) a rowing ergometer (Hiit Console, Core Health & Fitness LLC), (4) a cross trainer (Motion cross 600, Emotion Fitness), and (5) a step bench.

The participants then shifted to the bicycle ergometers. The bicycle training was initiated with 2 min start‐up bicycling, followed by 3 × 3‐min sequences of high‐intensity interval training constantly shifting between: 20 s at 85%–95% maximal workload and 20 s rest. The high‐intensity interval sequences were separated by two 3‐min steady‐state sequences. Participants were motivated by the supervising experienced physiotherapist to pedal at the pre‐calculated resistance and cadence levels according to their VO_2_ peak. Subsequent blood samples were collected within 2 min after end of exercise (ex02) and after 60 min (ex60). Participants only consumed water until the final blood sample. Capital Region's Ethics Board granted approval (H‐23006672).

Samples were transported to the sterile laboratory immediately after collection. Serum was processed and frozen within 1 h of being drawn. Serum samples were centrifuged at 1300*g* for 10 min; then aliquoted into 1.8‐mL cryotube ampoules for storage at −80°C.

### 2.2 Peripheral Blood Mononuclear Cell (PBMC) and Cancer Cell Lines

PBMCs were isolated using Lymphoprep density gradient medium (STEMCELL Technologies) from buffy coats obtained from the Rigshospitalet blood bank. PBMCs were then cryopreserved in foetal bovine serum (FBS, Gibco) + 10% dimethyl sulfoxide (DMSO, Honeywell) at −150°C.

The melanoma cell lines FM‐3 (ESTDAB‐007), FM‐45 (ESTDAB‐011), FM‐55‐M1 (ESTDAB‐012), FM‐82 (ESTDAB‐027), prostate cancer cell line PC‐3, and breast cancer cell line MDA‐MB‐231 were cultured in 1640 GlutaMAX‐I medium (RPMI, Gibco) supplemented with 10% FBS (R10). All cells were cultured at 37°C and 5% CO_2_.

### 2.3 CD3 T Cells Isolation and Cortisol Stimulation

PBMCs were thawed and rested overnight before isolation for CD3^+^ T cells using the MagniSort Human T Cell Enrichment Kit (Invitrogen) according to the manufacturer's instructions. Isolated CD3^+^ T cells were activated with plate‐coated anti‐CD3 antibodies (OKT3, eBioscience) or anti‐CD3/anti‐CD8–coated Dynabeads (Gibco) at a 1:1 bead‐to‐cell ratio in X‐vivo 15 media (Lonza) with 50 U/mL IL‐2 (Peprotech). Activated CD3^+^ T cells were stimulated with hydrocortisone (HC, cortisol, Tocris) at different concentrations ranging from 100 to 10 000 nM for 3–5 days before analysis. In the chronic setup, cortisol was kept in the culture until the end of the experiment. In the acute setup, cortisol was washed out after 4 h, and the culture was then replenished with X‐vivo 15 media with 50 U/mL IL‐2.

### 2.4 Proliferation Assay

Isolated CD3^+^ T cells were stained with CellTrace Violet (CTV, Life Technologies) at 37°C in the dark for 20 min. Cells were then incubated in X‐vivo 15 media with 5% human serum (Sigma‐Aldrich) for 5 min at room temperature to deactivate the CTV stain before activation and stimulation with cortisol. For analysis with flow cytometry, cells were harvested and washed with phosphate‐buffered saline (PBS) with 2% FBS (FACS buffer). The cell suspension was then stained with CD3‐Alexa Fluor 700 (UCHT1, BioLegend), CD8‐FITC (SK‐1, BD Biosciences), CD4‐PerCP‐Cy5‐5 (OKT4, BioLegend) and LIVE/DEAD Fixable Near‐IR Dead Cell Stain (Invitrogen) for 20–30 min at 4°C in the dark. The cells were then washed twice, resuspended in FACS buffer, and acquired on the NovoCyte Quanteon (Agilent).

### 2.5 Enzyme‐Linked Immunosorbent Assays (ELISA)

The levels of IFN‐γ and TNF‐α released in the cell culture supernatant were measured using ELISA assays (Invitrogen) based on the manufacturer's instructions. Cortisol levels from plasma and serum samples of healthy donors, as well as from cell culture supernatant, were measured using the Cortisol Parameter Assay Kit (R&D Systems) following the instructions of the manufacturer. Results were acquired using Epoch plate reader (BioTek) and analysed with Gen5 Take3 software (v1.00.4, BioTek).

### 2.6 xCELLigence Real‐Time Cell Analysis (RTCA)

To study the effect of cortisol on the proliferation of cancer cells and the cytotoxicity of T cells against cancer cells, the xCELLigence RTCA SP system was used. For the cancer cell growth assay, FM‐3 (15 000 cells/well), FM‐45 (5000 cells/well), FM‐55‐M1 (10 000 cells/well), FM‐82 (15 000 cells/well), PC‐3 (7500 cells/well) and MDA‐MB‐231 (5000 cells/well) cells were seeded in R10 in an E‐96 plate (Agilent). Suitable cell numbers are based on prior titration assays (data not shown). The plate was then incubated in the xCELLigence SP system (Agilent). Once the cells attached and started proliferating, cortisol at different concentrations or PBS as a vehicle were added. The plate was re‐inserted into the xCELLigence and the assay was continued for 72 h. The cells were analysed based on cell index that indicates the cell proliferation.

For the cytotoxicity assay, we used MAGE‐A3^a3a^ TCR specific CD3^+^ T cells that have been generated in our lab using lentiviral transduction of T cells from healthy donor PBMCs as described before [15]. Lentiviral vectors encoding high‐affinity MAGE‐A3^a3a^ TCR, along with the corresponding packaging and envelope plasmids (VSVG, REV, and gag/pol), were generously supplied by Adaptimmune Ltd. (Oxfordshire, UK) [16]. MAGE‐A3^a3a^ TCR transduced cells were treated with acute (4 h washout) or chronic cortisol at different concentrations for 3 days in 100 U/mL IL‐2. The day before the co‐culture, FM‐55 M1 cells were plated at 10 000 cells/well in the E‐96 plate and inserted into the xCELLigence SP system. After 25–28 h, MAGE‐A3^a3a^ specific T cells previously treated with cortisol were washed and added to the plate at different E:T ratios titrated from 10:1 to 1:1. The cell index was then measured for 72 h. To calculate the cytotoxicity of T cells, a condition with cancer cells alone was included. The cell index of cancer cells was normalised to the values of the cancer cells alone for each condition and converted into percentage of cytolysis. Each condition was run in triplicates. The RTCA software Basic 2.0 (Agilent) was used for data acquisition.

### 2.7 Intracellular Staining

MAGE‐A3^a3a^ TCR specific CD3^+^ T cells were stained intracellularly for degranulation markers, cytokines, and glucocorticoid receptors upon activation by co‐culturing with FM‐55 M1 cancer cells at an effector‐to‐target (E:T) ratio of 1:3. For positive control, 5 ng/mL phorbol myristate acetate (PMA, Sigma Aldrich) and 75 nM ionomycin (Sigma Aldrich) were added. For negative control, cells were left untreated with X‐vivo 15 media. All cell samples were incubated with anti‐CD107a (H4A3, BD Biosciences) and Brefeldin A (Biolegend) for 5 h incubation at 37°C. For surface staining, cells were then incubated with anti‐CD3‐ Alexa Fluor 700 (UCHT1, BioLegend), CD8‐BV605 (SK‐1, BioLegend), CD4‐BV711 (OKT4, BioLegend) and LIVE/DEAD Fixable Near‐IR Dead Cell Stain (Invitrogen) for 20–30 min at 4°C in the dark. After that, the cells were washed twice with FACS buffer, then fixed and permeabilised with Intracellular Fixation and Permeabilisation Buffer Set (eBioscience) overnight at 4°C. Subsequently, cells were washed with 1xPermeabilisation Buffer (eBioscience) and stained with intracellular antibody mix as described for surface staining. The intracellular antibodies are the following: IFN‐γ‐BV510 (4S.B3, BioLegend), TNF‐α‐PE‐CF594 (Mab11, BD Biosciences), Granzyme B‐APC (GB‐11, Fisher Scientific) and glucocorticoid receptor‐FITC (5E4, Fisher Scientific). Lastly, the cells were washed with 1xPermeabilisation Buffer and resuspended in FACS buffer before analysis on the NovoCyte Quanteon.

### 2.8 Phenotyping With Flow Cytometry

Phenotypes of MAGE‐A3^a3a^‐specific T cells following cortisol treatment were characterised by flow cytometry. Surface markers were stained using the methods described above with the following antibodies: CD3‐Alexa Fluor 700 (UCHT1, BioLegend), CD8‐APC (RPA‐T8, BD Biosciences), CD4‐BV711 (OKT4, BioLegend), PD‐1‐PE (eBioJ105, Thermo Fisher), TIM‐3‐BV421 (7D3, BD Biosciences) and LIVE/DEAD Fixable Near‐IR Dead Cell Stain (Invitrogen). All flow cytometry data were analysed using FlowJo v10.

### 2.9 Statistical Analysis

Statistical analyses included two‐tailed paired *t*‐tests for two‐group comparisons and one‐way ANOVA followed by Dunnett's multiple comparisons test for multiple groups. Multiple comparisons were used to compare each condition to the control, within acute or chronic conditions separately. Results were expressed as means ± SEM. Data analysis was performed using GraphPad Prism 10.1.2. *p* values less than 0.05 were statistically significant; *p ≤ 0.05, **p ≤ 0.01, ***p ≤ 0.001 and ****p ≤ 0.0001, not significant when *p* > 0.05.

## 3 Results

### 3.1 Cortisol Release Into Circulation Is Influenced by the Duration of Exercise

**FIGURE 1:**
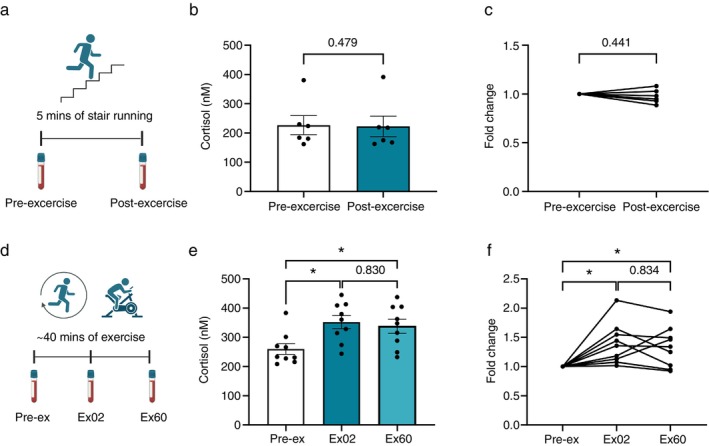
Release of cortisol is dependent on duration of exercise. (a) Illustration of the running setup, where a blood sample was collected before (pre‐exercise) and within 2 min after (post‐exercise) 5‐min stair and ground running. Created with Biorender. (b) The concentration of cortisol (nM) in plasma of healthy donors, pre‐exercise, and post‐exercise measured by ELISA (*n* = 6). (c) The fold change of cortisol levels in plasma after 5‐min sprinting (*n* = 6). (d) Illustration of INHALE exercise regimen, where a blood sample was collected before (pre‐ex), within 2 min post (ex02), and 60 min post (ex60) 40 min of high‐intensity interval training (HIIT). Created with Biorender. (e) The concentration of cortisol (nM) pre‐ex, ex02, and ex60 measured in serum by ELISA (*n* = 9). (f) Fold change of cortisol levels relative to pre‐ex (*n* = 9). Data is representative of four independent experiments. Statistical analysis was performed by paired *t*‐test (b, c) and one‐way ANOVA (e, f). Error bars represent mean ± SEM. *p ≤ 0.05, not significant when *p* > 0.05.

Exercise is a form of acute stress that can trigger the increased release of cortisol [17]. To understand the impact of cortisol on T cell function, we first determined how exercise affects the level of cortisol in healthy adults. Blood samples of healthy volunteers were collected before and within 2 min after stair and ground running for 5 min (Figure 1a). Their plasma levels of cortisol were measured by ELISA. We observed no difference between pre‐exercise and post‐exercise levels of cortisol after 5 min of sprint (Figure 1b,c). We also investigated the serum cortisol levels in the participants of the INHALE trial before, within 2 min, and 60 min after completing 40 min of high‐intensity exercise (Figure 1d). We saw that after cessation of exercise (ex02), the level of cortisol increased significantly compared to pre‐exercise levels (pre‐ex). Notably, the levels remained significantly elevated even 60 min (ex60) after the exercise session had ended (Figure 1e,f). Since we only saw an increase in cortisol from INHALE participants, it suggests that prolonged high‐intensity exercise, but not short sprint, provokes increased cortisol levels. These findings highlight the role of duration of exercise in regulating the increase in circulating levels of cortisol.

### 3.2 Glucocorticoid Receptors Are Stably Expressed in Chronic Cortisol Condition

Stress‐induced cortisol can have both promoting or suppressing effects on the immune responses depending on the dose and duration of signalling through GCRs [13]. Cortisol increased by acute stress like exercise is suggested to be transient, whereas psychological stress leads to chronic cortisol release [18, 19]. While exercise has a positive impact on the prognosis of several cancers, elevated cortisol levels associated with chronic stress have been suggested to promote tumourigenesis and cancer progression, possibly in part due to immune‐mediated changes [7, 20]. Therefore, we studied how the duration of cortisol signalling affects T cell functions by establishing acute and chronic cortisol stimulation. We first assessed the stability of cortisol in vitro by adding 1000 nM cortisol to culture media alone or with cells. The level of cortisol was measured at different time points, which showed that cortisol was stable for up to 6 days regardless of the presence of cells (Figure S1a). Furthermore, adding cortisol twice a day for 4 days resulted in accumulation in cortisol levels over time, unaffected by cell presence (Figure S1b), suggesting that a single dose of cortisol was sufficient to induce chronic stimulation.

**FIGURE 2:**
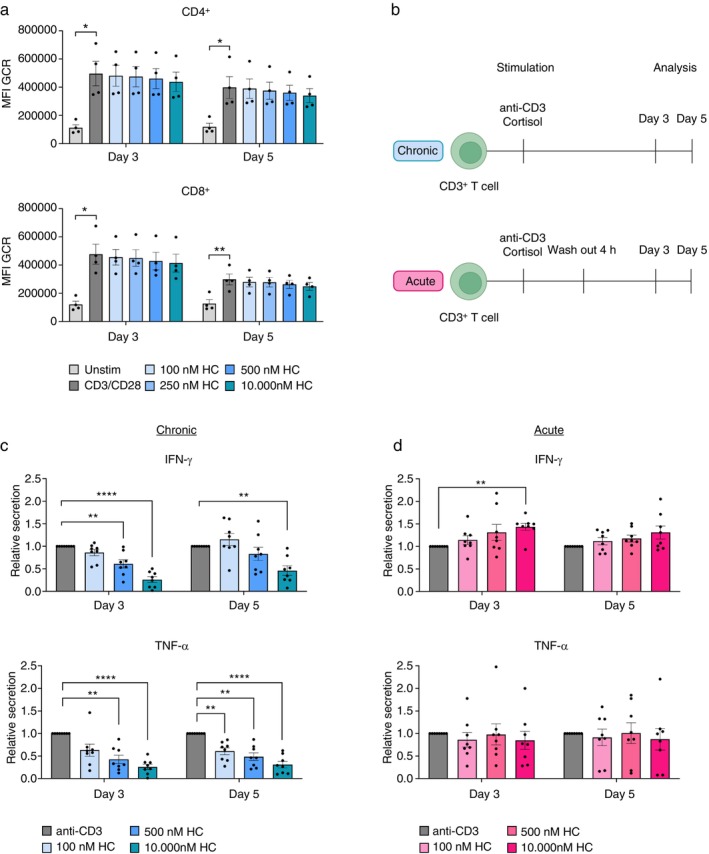
Chronic cortisol do not alter glucocorticoid receptors and decreases cytokine production of activated CD3^+^ T cells. (a) GCR expression (MFI) on CD4^+^ and CD8^+^ T cells 3 and 5 days after stimulation with cortisol at different doses (*n* = 4). (b) Acute and chronic treatment of activated CD3^+^ T cells with cortisol. Created with Biorender. Relative levels of IFN‐γ and TNF‐α in culture supernatant from anti‐CD3‐activated CD3^+^ T cells 3 and 5 days in the presence or absence of (c) chronic or (d) acute cortisol (*n* = 8). Error bars represent mean ± SEM. *p ≤ 0.05, **p ≤ 0.01, ***p ≤ 0.001, not significant when *p* > 0.05. HC: cortisol.

According to the literature, almost all cell types in the body express GCRs [3]. Prolonged exposure to cortisol due to chronic stress may alter GCR expression and result in GCR desensitisation that possibly disrupts the regulation of inflammatory responses [21]. Thus, to explore the impact of cortisol on T cell functionality, we studied how cortisol influences the expression of GCR on T cells. We activated human T cells with CD3/CD28 beads in the absence or presence of cortisol and quantified the GCR expression after 3 and 5 days. Concentrations of cortisol were based on baseline (100 nM), low (250 nM) and high (500 nM) physiological levels as well as high dose (10 000 nM) that are frequently used in vitro. Our data showed that both CD4^+^ and CD8^+^ T cells expressed the GCR, and that the expression significantly increased upon activation (Figure 2a). GCR expression level was not affected by continuous exposure to cortisol at any concentration, neither on Day 3 nor 5. This indicates that the expression of GCR on activated T cells is stable and not downregulated by prolonged cortisol exposure.

### 3.3 Chronic, but not acute, cortisol exposure suppresses T cell cytokine production and proliferation

Our findings suggest that we can mimic chronic cortisol signalling in vitro with one dose of cortisol without interfering with the GCR expression on T cells. To establish acute stimulation, we washed out cortisol after 4 h of stimulation, which is the time suggested for cortisol to return to baseline levels after exercise [19]. We then examined the long‐term impact of acute and chronic cortisol on cytokine release. Activated CD3^+^ T cells were stimulated with increasing concentrations of cortisol, acutely or chronically, and the supernatant was collected after 3 and 5 days for ELISA (Figure 2b). We saw that chronic stimulation with cortisol inhibited both IFN‐γ and TNF‐α secretion levels in a dose‐dependent manner after 3 days (Figure 2c). This dose‐dependent effect of chronic stimulation was also observed on day 5 though the IFN‐γ levels seemed to recover under cortisol treatment at physiological concentrations. In contrast, acute exposure to cortisol did not affect the levels of IFN‐γ and TNF‐α in the long‐term, except for a slight increase with the highest cortisol concentration (Figure 2d).

**FIGURE 3:**
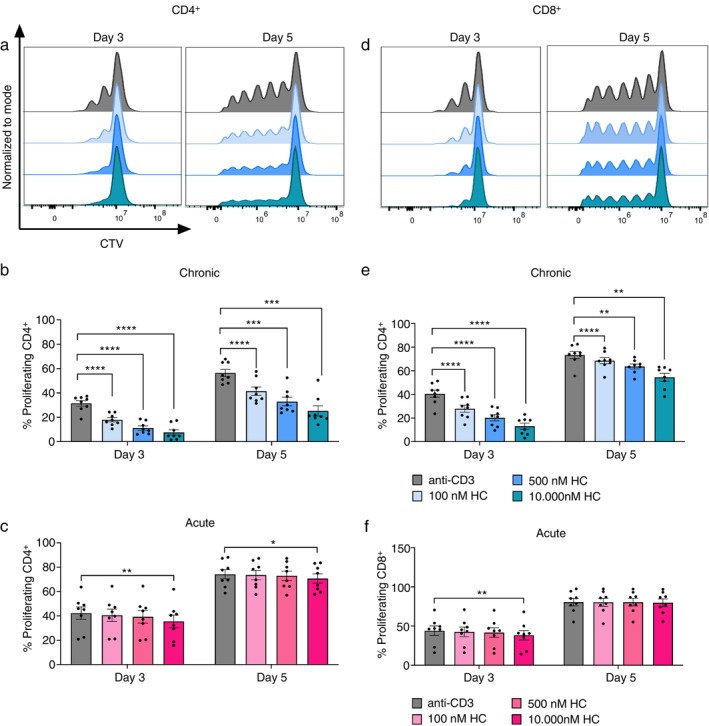
Chronic cortisol decreases the proliferation of activated CD3+ T cells in a dose‐dependent manner. Isolated CD3^+^ T cells were activated with plate coated anti‐CD3 in the absence or presence of chronic (a–c) or acute (d–f) cortisol for 3 or 5 days. Representative histograms of proliferating CD4^+^ (a) and CD8^+^ (d) T cells under chronic cortisol. The proliferation of CD4^+^ (b, c) and CD8^+^ (e, f) T cells is shown as percentage of dividing cells (*n* = 8). Proliferation was quantified by CTV dilution and analysed by flow cytometry. Error bars represent mean ± SEM. *p ≤ 0.05, **p ≤ 0.01, ***p ≤ 0.001, ****p ≤ 0.0001, not significant when *p* > 0.05. HC: cortisol.

We further investigated the effect of cortisol on the proliferation of T cells. After chronic treatment, we saw that cortisol inhibited the proliferation of both CD4^+^ (Figure 3a,b) and CD8^+^ T cells (Figure 3d,e). Notably, the inhibitory effect was greater with increasing concentrations of cortisol. The effect was seen on both Day 3 and 5, which is consistent with the stable expression of GCRs and cortisol levels during cortisol treatment. We could also exclude that the inhibitory effect of cortisol was due to toxicity since the cell viability was similar for all conditions (Figure S2a,b). On the contrary, acute exposure to cortisol for 4 h did not have a long‐term impact on the proliferation of the studied T cell subtypes (Figure 3c,f). Though the highest cortisol concentration at 10 000 nM slightly decreased the percentage of dividing CD4^+^ and CD8^+^ T cells on Day 3, it was largely recovered on Day 5. Our data suggested that chronic cortisol signalling, in contrast to acute stimulation, has a prolonged inhibitory impact on T cell proliferation and cytokine secretion.

### 3.4 Chronic Cortisol Enhanced the Growth of Several Cancer Cell Lines

**FIGURE 4:**
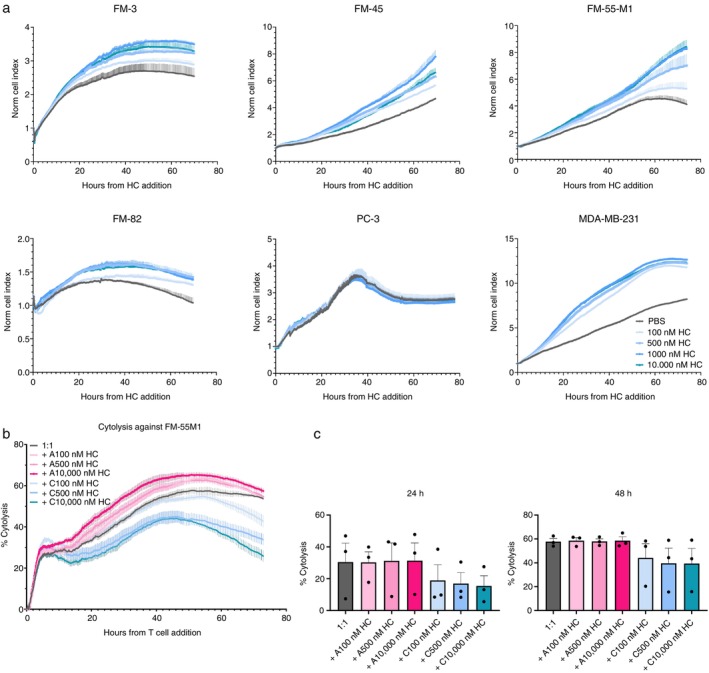
Chronic cortisol promotes the growth of the cancer cells while inhibiting killing activity of T cells. (a) Proliferation of FM‐3, FM‐45, FM‐55 M1, FM‐82, PC‐3 and MDA‐MB‐231 reflected by the cell index during 72 h after adding cortisol in the xCELLigence system. (b) Representative cytolysis plot comparing the killing ability of MAGE‐A3^a3a^‐specific T cells against FM‐55 M1 (grey) after pretreatment with acute (pink) or chronic (blue) cortisol at different concentrations for 3 days. Percentage of cytolysis was calculated at the E:T ratio of 1:1. (c) Comparison of cytolysis of cortisol‐treated MAGE‐A3^a3a^‐specific T cells after 24 and 48 h co‐cultured with FM‐55 M1 (*n* = 3). All the conditions were run in triplicates. Error bars represent mean ± SEM. A: acute; C: chronic; HC: cortisol.

Chronic stress has previously been suggested to be a risk factor of cancer growth and progression [22]. However, stress is mediated by the release of different hormones, including catecholamine and cortisol [4]. Therefore, we wanted to study the direct effect of cortisol alone on the growth of cancer cells. We used the xCELLigence system to examine the growth of six different cancer cell lines under treatment with different concentrations of cortisol. We saw that cortisol at different doses promoted the growth of the melanoma cell lines FM‐3, FM‐45, FM‐55‐M1 and FM‐82, and the breast cancer cell line MDA‐MB‐M1 compared to the negative control (Figure 4a). The effect remained up to 72 h after the addition of cortisol, indicating that chronic exposure to cortisol alone has a long‐term promoting effect on cancer cell growth. The only exception was with the prostate cancer cell line PC‐3, where cortisol did not seem to have an effect.

### 3.5 Chronic Cortisol Diminished T Cell Cytotoxicity Compared to Acute Cortisol

We then determined how acute cortisol affects the killing activity of T cells against cancer cells compared to chronic cortisol. Tumour‐specific T cells generated using lentiviral transduction with the high‐affinity MAGE‐A3^a3a^ TCR were co‐cultured with the FM‐55 M1 cell line that expresses the MAGE‐A3 cancer testis antigen [15]. MAGE‐A3^a3a^ TCR‐transduced T cells were pre‐treated with acute (4‐h washout) or chronic cortisol for 3 days, then washed to remove excess cortisol that may have a direct impact on cancer cells. xCELLigence data showed that chronic pre‐exposure to cortisol attenuated the killing ability of T cells, even after cortisol was removed from the culture (Figure 4b). The effect was more pronounced with the high concentrations of cortisol. Over the same 72‐h time course, T cells acutely exposed to cortisol showed no clear difference in cytotoxicity compared to the non‐treated control. When comparing the cytolysis at different time points during the assay, chronic cortisol at all concentrations showed a trend to reduce the killing of cancer cells at 24 and 48 h compared to acute cortisol (Figure 4c).

**FIGURE 5:**
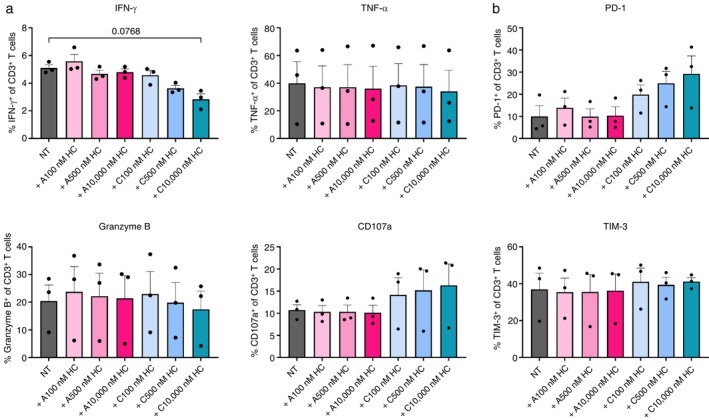
Cortisol‐treated T cells shows a trend toward reduced release of cytotoxic molecules and increased PD‐1 expression. MAGE‐A3^a3a^ TCR transduced T cells were either left non‐treated (NT) or treated with acute or chronic cortisol for 3 days before flow analysis. (a) Cytokine expression and degranulation of T cells after 5 h co‐cultured with FM‐55 M1 (E:T ratio of 1:3) (*n* = 3). (b) Expression levels of exhaustion markers PD‐1 and TIM‐3 on inactivated T cells on day 3 (*n* = 3). All the conditions were run in triplicates. Error bars represent mean ± SEM. A: acute; C: chronic; HC: cortisol.

To further assess the reactivity of cortisol‐treated T cells, we investigated the release of cytokines and cytolytic molecules using an intracellular staining assay. MAGE‐A3^a3a^‐specific T cells pre‐treated with acute and chronic cortisol were cultured with FM‐55 M1 cells for 5 h and stained for intracellular cytotoxic and cytokine markers. We observed a tendency of decreased IFN‐γ expression with increasing cortisol concentrations, and a slight decrease of Granzyme B at high‐dose cortisol in the chronic setup (Figure 5a). The chronic treatment did not show a difference in TNF‐α expression but slightly increased the expression of the degranulation marker CD107a. Thus, cells under chronic cortisol exposure seemed to degranulate to a higher extent, but supposedly with fewer Granzyme B molecules in the granules than the non‐treated cells. Additionally, chronic cortisol‐treated T cells displayed an increasing trend in the expression of the activation/exhaustion marker PD‐1, but not TIM‐3 (Figure 5b). Four hour exposure to cortisol did not show a difference in any of the studied markers, indicating no effect of acute cortisol signalling on T cell reactivity.

## 4 Discussion

Exercise has been found to promote mobilisation of immune cells, especially T and NK cells [23], which infiltrate tumours and correlate with restricted tumour growth in mice [9, 10]. These findings proposed exercise as a potential tool to increase the efficacy of immunotherapies [7]. However, exercise triggers numerous physiological processes, and among all the exercise‐induced immunomodulators, cortisol seems to have contrasting effects on immune cells in different contexts. We therefore studied the connection between cortisol and physical activity, as well as the impact of cortisol on T cell function and cancer cell growth.

Cortisol is one of the signalling molecules responsible for exercise‐induced leukocytosis [19, 24]. The increased cortisol levels during exercise have been suggested to be mediated by IL‐6 and increase the number of circulating neutrophils [19, 25]. We found that compared to 40‐min high‐intensity exercise, 5 min of sprinting running was not sufficient to provoke an increase in cortisol plasma levels in healthy participants. This finding indicates that the duration of exercise possibly determines the release levels of cortisol and how long the cortisol levels persist. The magnitude of cortisol release may also be affected by intensity, as a previous study has shown a significant increase in cortisol levels with 30 min at 60%–80% VO_2_ max but not at 40% [26]. However, it is possible that 5‐min running induced the increase in cortisol levels in a delayed fashion. Thuma et al. found that the serum levels of cortisol were only clearly higher between 20 and 40 min after the onset of exercise and peaked after approximately 60 min [27]. This is consistent with our finding that 5‐min running was sufficient to increase catecholamine levels (data not shown), which is known to be released more rapidly [19, 24]. Therefore, to fully understand the dynamics of cortisol release, later time points should also be included.

While the circulating half‐life of endogenous cortisol is estimated to range from 60 to 115 min [28], cell culture media lack the enzymes necessary for cortisol inactivation or degradation [29]. To our understanding, there is no study following the stability of cortisol in vitro. We found that the cortisol concentrations remained unchanged within 6 days, underscoring the much higher stability of cortisol in vitro compared to in vivo. Nonetheless, GCR expression has been suggested to be affected by chronic exposure to cortisol that may result in GCR resistance [21]. Our in vitro setup of chronic cortisol did not impair GCR expression on activated T cells, confirming the feasibility of this model to study cortisol impact on T cells. Notably, GCR expression on CD4^+^ and CD8^+^ is upregulated upon activation. This is supported by Acharya et al., who showed that tumour‐antigen specific CD8^+^ tumour‐infiltrating lymphocytes (TILs) expressed higher levels of GCRs, indicating enhanced GC signalling following TCR activation [30].

Much of our knowledge about GCs comes from studies using potent, long‐acting synthetic GCs such as dexamethasone or prednisone for their immunosuppressive effects [13]. In contrast, hydrocortisone (natural cortisol) is considered less potent, more relevant to psychological distress, and has been suggested to have both potentiating or inhibitory effects on T cell immunity depending on the dose and duration of cortisol signalling [13, 14, 29]. To the best of our knowledge, no study has tried to discern the impact of acute and chronic cortisol in a controlled condition. Our data revealed that exposure to cortisol for only 4 h did not reduce IFN‐γ and TNF‐α secretion but rather slightly increased IFN‐γ at the highest dose for up to 5 days after stimulation. Conversely, chronic cortisol decreased IFN‐γ and TNF‐α dose‐dependently. This data aligns with previous findings on human and murine T cells showing reduced IFN‐γ and TNF‐α release after treatment with dexamethasone—a synthetic GC [30, 31, 32].

Similarly, continuous exposure to cortisol suppressed the proliferation of both CD4^+^ and CD8^+^ T cells. The inhibitory effect of GCs on T cell proliferation and cytokine production may be due to the downregulation of several transcription factors such as AP‐1, NF‐κB and NFAT involved in TCR signalling and T cell activation [33, 34, 35]. This in turn inhibits the transcription of proinflammatory genes [36, 37] and prevents the proliferation of T cells [35, 38]. The suppression of proliferation was not observed in our acute setup despite a slight decrease at the highest dose. Our findings suggest that transient elevation in cortisol levels, such as during exercise, was not sufficient to induce long‐term damage on the activation and function of T cells.

Chronic stress promotes cancer development not only by impairing antitumor immunity but also through direct action on cancer cells [4]. Studies have shown contradictory effects of stress‐induced cortisol on tumour progression depending on the cancer type. For instance, while GCs are used to induce apoptosis in haematological malignancies treatment [39], they increased the expression of anti‐apoptotic genes in human epithelial cancers [40]. GCs were also shown to induce cancer proliferation at lower doses, while suppressing it at high doses [41]. Here we saw that cortisol at both physiological and pharmacological concentrations enhanced the tumour growth in vitro of different melanoma and breast cancer cell lines. This agrees with previous in vitro studies demonstrating the promoting effects of GCs on human cancer cells [42, 43, 44].

Although some studies suggested that GCs inhibited antitumour immunity and blocking this signalling pathway restored tumour growth control in vivo, little is known about their impact on T cell cytotoxicity [5, 30]. Using the xCELLigence system to monitor T cell cytolysis in real‐time for 72 h, we found no impairment in the killing activity of antigen‐specific T cells pre‐treated with acute cortisol, while chronic cortisol exposure decreased cytotoxicity. Our findings contrast with previous in vivo data showing that GCs did not affect the antitumour activity of activated CD8^+^ T cells, likely due to the dynamic interaction of cells in the TME, many of which express GCRs [45]. However, our results align with another study on TILs demonstrating the impairment of T cell killing activity following GC treatment, which could be restored after removing GCs and resting the cells for 3 days [46]. The dampened killing ability was associated with a tendency for downregulation in IFN‐γ and Granzyme B expression, but not in the acute conditions. Despite the increased CD107a expression, the killing ability of antigen‐experienced T cells was suggested to be mediated by the granule contents rather than degranulation activity [47]. Therefore, even with increased degranulation, the impaired killing may result from the lower Granzyme B levels in the granules and reduced IFN‐γ release. The induction of PD‐1 by chronic cortisol is also consistent with previous findings in activated T cells [30, 48], while the impact of short‐term exposure was not reported elsewhere. Together, our data suggest that cortisol in the chronic context inhibits T cell effector functions, while acute exposure does not inflict long‐term damage.

Overall, our data demonstrated the contrast in the impact of cortisol signalling depending on different durations of action. This raises the caution when using synthetic GCs for treatment‐related inflammation in cancer patients, who already experience elevated cortisol levels due to chronic distress [5]. Chronic GC‐induced immune suppression could potentially be reversed by inhibition of GCR using genetic modification or GCR antagonists such as mifepristone. This has been supported by recent in vitro and in vivo studies, which offered potential approaches to improve adoptive immunotherapies and cancer treatment [5, 30, 49]. Our study also underscores the importance of considering intensity and duration when designing exercise programmes as an adjuvant cancer therapy. Using bouts of exercise with sufficient intensity could induce short‐term cortisol release that promotes leukocytosis. Such short‐lived cortisol levels are not expected to exacerbate negative impacts of chronic stress‐induced cortisol on antitumour immunity, which could be at the same time abolished by blocking GCR signalling.

## Author Contributions

T.V.L., L.F.H. and T.S. performed the experiments; T.V.L. and L.F.H. analysed the results; K.L. performed the trial and provided clinical materials; T.V.L., L.F.H., P.t.S. and G.H.O. designed the research and interpreted the data; P.t.S. and G.H.O. supervised the project; T.V.L. wrote the manuscript with input and approval from all the coauthors.

## Conflicts of Interest

The authors declare no conflicts of interest.

## Supporting information


**Data S1:** Supporting Information.

## Data Availability

The data that support the findings of this study are available from the corresponding author upon reasonable request.
