# Supplementary material for: Exercise Duration Modulates Cortisol Release and Chronic Cortisol Exposure Jeopardises T Cell Effector Functions
Source: Immunology. 2025 Aug 12;177(1):94–105. doi: 10.1111/imm.70028 (PMC12665805; doi:10.1111/imm.70028)
Supplement: Supplementary file 1 — Data S1: Supporting Information. [file IMM-177-94-s001.docx]

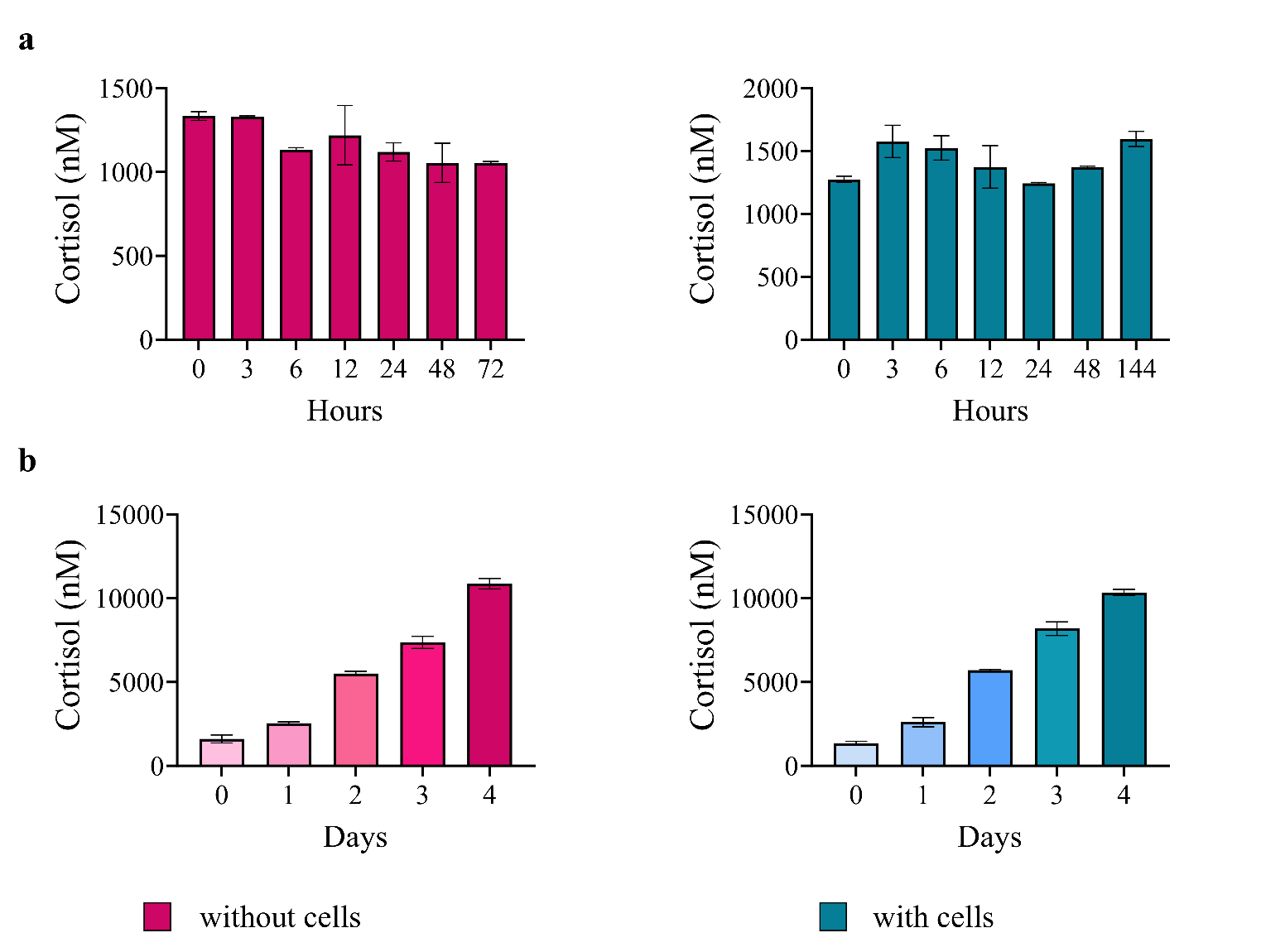


**Supplementary Figure 1 Chronic cortisol is highly stable *in vitro***

Cortisol levels at different timepoints after adding **a)** a single dose of cortisol at 1,000 nM or **b)** 1,000 nM cortisol twice a day for 4 days to cell culture media in the absence (pink) or presence (blue) of PBMCs. The supernatant was collected right after addition of the first dose (0) of cortisol, as well as every day before adding the next dose of cortisol (day 1-4). Cortisol levels were measured by ELISA. Error bars represent mean ± SEM.


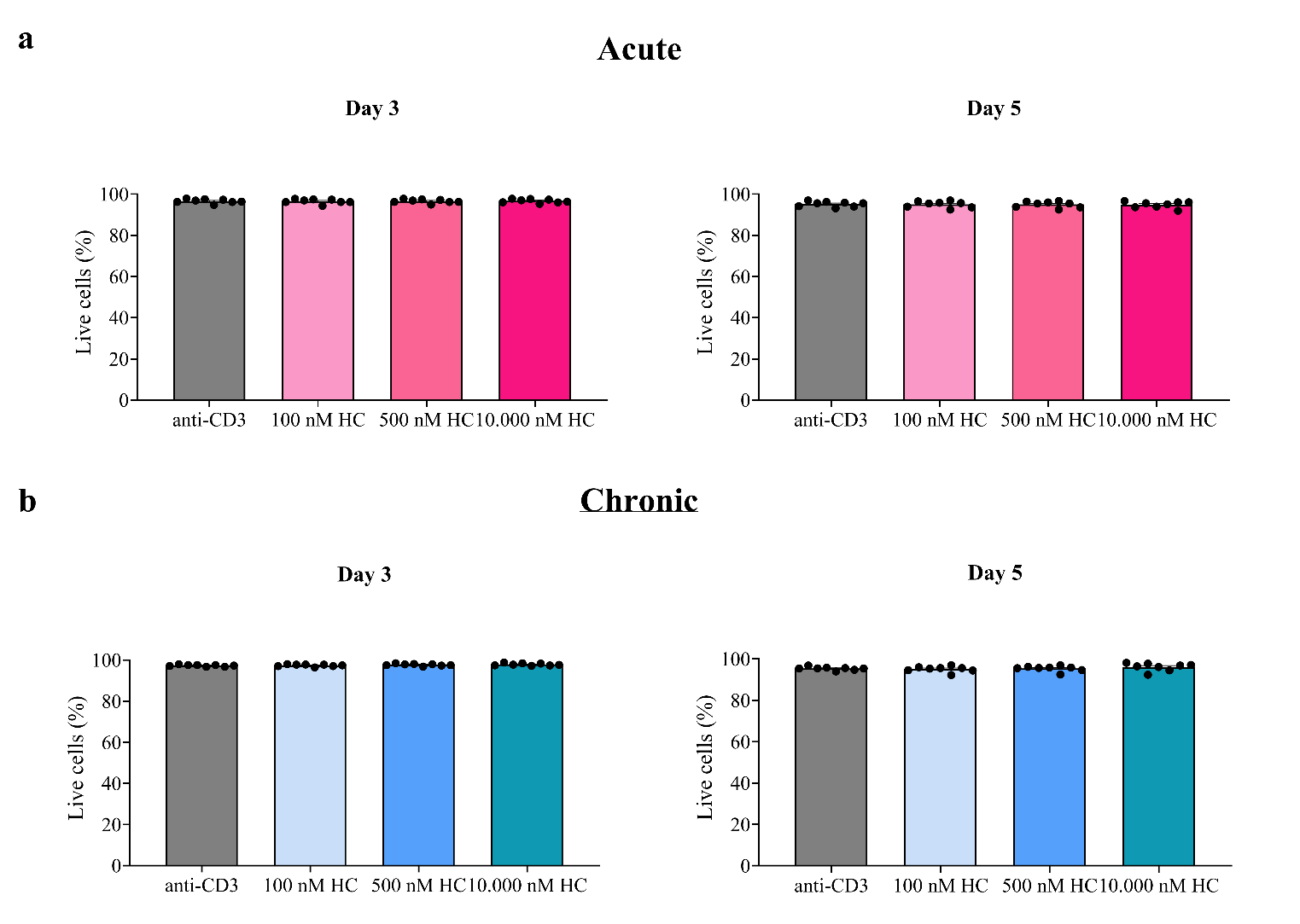


**Supplementary Figure 2 T cell viability is not dampened by acute and chronic treatment with cortisol upon activation**

CD3+ T cells activated with anti-CD3 with or without **a)** acute and **b)** chronic exposure to cortisol was analyzed for viability together with proliferation assay on day 3 and day 5. (n=8).
